# Trait-specific tracking and determinants of body composition: a 7-year follow-up study of pubertal growth in girls

**DOI:** 10.1186/1741-7015-7-5

**Published:** 2009-01-26

**Authors:** Sulin Cheng, Eszter Völgyi, Frances A Tylavsky, Arja Lyytikäinen, Timo Törmäkangas, Leiting Xu, Shu Mei Cheng, Heikki Kröger, Markku Alèn, Urho M Kujala

**Affiliations:** 1 Department of Health Sciences, University of Jyväskylä, Jyväskylä, Finland; 2 Department of Preventive Medicine, University of Tennessee Health Science Center, Knoxville, Tennessee, USA; 3 Department of Orthopaedics and Traumatology, Kuopio University Hospital, Kuopio, Finland; 4 Department of Medical Rehabilitation, Oulu University Hospital and Institute of Health Sciences, University of Oulu, Oulu, Finland

## Abstract

**Background:**

Understanding how bone (BM), lean (LM) and fat mass (FM) develop through childhood, puberty and adolescence is vital since it holds key information regarding current and future health. Our study aimed to determine how BM, LM and FM track from prepuberty to early adulthood in girls and what factors are associated with intra- and inter-individual variation in these three tissues.

**Methods:**

The study was a 7-year longitudinal cohort study. BM, LM and FM measured using dual-energy X-ray absorptiometry, self-reported dietary information, leisure time physical activity (LTPA) and other factors were assessed one to eight times in 396 girls aged 10 to 13 years (baseline), and in 255 mothers once.

**Results:**

The location of a girl's BM, LM and FM in the lower, middle or upper part of the sample distribution was established before puberty and tracked in its percentile of origin over 7 years (*r *= 0.72 for BM, *r *= 0.61 for LM, and *r *= 0.65 for FM all *p *< 0.001 first vs. last measurements' ranking). Seventy-three percent of those in the lowest quartile for BM and 69% for LM, and 79% of those in the highest quartile for FM at baseline remained in their quartile at 7-year follow-up. Heritability was estimated to contribute 69% of the total variance of the BM, 50% of the LM, and 57% of the FM. Besides body size, diet index (explaining 9% of variance), breast feeding duration (6%) and mother's BM (9%) predicted high BM. Diet index and high LTPA predicted high LM (24% and 14%, respectively), and low FM (25% and 12%, respectively), and low level of parental education predicted high FM (4%).

**Conclusion:**

Individual levels of BM, LM and FM are established before puberty and track in a trait-specific manner until early adulthood. Girls who are prone to develop low BM and LM and high FM in adulthood can be identified in prepuberty. The developments of three components of body composition are inter-related during growth. BM was the most heritable trait while LM the most environmentally modifiable. Diet and physical activity played an important role in increasing LM and preventing the accumulation of excessive FM.

## Background

The biology of bone, muscle and fat tissues and their associated disorders such as obesity, osteoporosis and sarcopenia are closely inter-related and share several common origins, including genetic and environmental factors [1-5]. Skeletal muscle, acting in conjunction with energy expenditure and related hormone regulation, is one of the major tissues responsible for metabolic status [4-6]. As muscle and adipose tissue are closely linked anatomically, biologically and pathologically, study of the interrelationship between these two tissues is of great importance in understanding the pathogenesis and development of diseases related to obesity and physical activity/inactivity [6-8]. Furthermore, leptin, an adipocyte-derived hormone, is a major regulator of bone remodelling [9]. A recent finding in an animal study showed that bone exerts an endocrine regulation of energy homeostasis [2]. Together these studies emphasized that when studying the development of body composition, bone mass (BM), lean mass (LM), and fat mass (FM) should be considered together.

Changes in BM, LM and FM during puberty are indicators of metabolic processes, and thus hold pertinent information regarding current and future health [10]. Although it is well known that the components of body composition track, especially from adolescence onwards [10], there is a paucity of information on how bone, muscle and fat tissues change during the rapid phase of adolescent growth, and when non-optimal body composition characteristics can be detected.

Early identification of the risk factors for future diseases is the first step towards effective prevention strategies. In order to set up cost-effective programmes promoting healthy growth in body composition, more attention should be paid to what factors contribute to intra- and inter-individual variation in BM, LM and FM during adolescent growth, and in what ways.

In this longitudinal study, we examined the development of three components of body composition during growth and attempted to answer the following questions: i) Do BM, LM and FM track, and if so, how are they inter-related from prepuberty to early adulthood? ii) Can girls who are prone to develop low BM and LM and high FM in adulthood be identified in prepuberty? iii) What factors are associated with intra- and inter-individual variation in these three components of body composition?

## Methods

### Study population

The subjects were first contacted via class teachers teaching grades 4 to 6 (age 9 to 13 years old) in 61 schools in the city of Jyväskylä and its surroundings in Central Finland (96% of all the schools in these areas). Briefly, of the eligible subjects, 396 girls participated in the laboratory tests one to eight times during a maximum period of 8 years (mean duration of total follow-up was 7.5 years and mean age at last follow-up was 18.3 years; *n *= 396 at baseline, *n *= 208 at 6-month, *n *= 201 at 12-month, *n *= 191 at 18-month, *n *= 220 at 24-month, *n *= 88 at 36-month, *n *= 61 at 48-month, and *n *= 236 at 84-month follow-up). Of the 396 girls, 258 participated in a calcium and vitamin D intervention during the first 2 years [11]. Although no intervention effects on body composition were found, whether or not they were in the intervention group was taken into account in the present analysis. The study protocol was approved by the ethical committee of the University of Jyväskylä, the Central Finland health care district, and the Finnish National Agency of Medicines. Informed consent was given by all subjects and their parents prior to the assessments.

In addition, 255 mothers (age 32 to 58.9 years), 79 sisters (age 10 to 31 years), and 41 brothers (age 9 to 31 years) participated in the same study procedures as the girls. Among mothers, data on 144 premenopausal mothers (mean age 45.0, range 32 to 54 years) were used for comparison with their daughters at age 18 and to estimate the maternal contribution to BM, FM, and LM. The sibling data were used in estimation of the heritability in the broad sense. The participants provided their written consent in accordance with the guidelines laid down by the ethical committees.

### Background information

All the information on all participants was collected and laboratory tests performed within two weeks' period during the same month of the year throughout the 7-year follow-up to avoid seasonal effects.

Lifestyle and behavioural characteristics as well as medical history were collected via a self-administered questionnaire. Girls under age 15 filled in the questionnaire with their parents' assistance, and all the questionnaires were checked by a study nurse. Breast feeding was expressed in months. The socio-economic factors included the highest level of parental education, how wealthy the family is, and whether the girl was living with both parents or a single parent (see Additional files 1 and 2). Birth crown-heel length and birth weight were obtained from the girl's growth chart.

Dietary information was obtained from a food-intake diary kept for three days (two ordinary school days and one weekend day, see Additional file 3) as described elsewhere [12]. The food records contained time of eating, items and portion sizes. The families were given written instructions and an example of how to record food consumption with a help of portion guidebook. Details of all foods and drinks including the type and commercial brand name were filled in the records. In addition, the type of diet (if restricted) was requested in a separate question. During the coding process the food records were checked by the nutritional students. Dietary intakes were analyzed using Micro-Nutrica software (version 2.5), Finland. The program has been validated and gives a reasonably good estimate of the intake of energy and most nutrients compared with chemical analyses of the diet [13]. For this report, on the basis of earlier studies [14-17], the nutrients related to body composition were chosen to compute a dietary intake index, including intakes of protein (g/day), calcium (Ca) (mg/day), potassium (K) (mg/day), phosphorus (P) (mg/day) and magnesium (Mg) (mg/day) (for more details, see statistical methods).

A leisure time physical activity (LTPA) score was calculated on the basis of questionnaire (see Additional files 1 and 2) [18] responses as follows: the metabolic equivalents of the girl's three favourite leisure time exercises (outside school) during the previous six months × duration of each exercise session × the weight-bearing condition of each exercise (for example, non-weight-bearing = 1 and weight-bearing = 2, as weight-bearing exercise is important for bone development) × times/week of participation of those exercises. The score was validated against a 7-day physical activity diary and heart rate monitor for estimating energy expenditure in a sub-group; Bland-Altman analysis showed an acceptable agreement.

### Anthropometrical and maturation assessments

Body height (cm) and weight (kg) were measured using standardized protocols and body mass index (BMI = kg × m^-2^) was calculated. Sexual development was determined according to Tanner grading system by a nurse [19]. The age of menarche was defined as the first onset of menstrual bleeding and was collected by questionnaire, retrospective phone call, and/or interview during a clinical visit.

### Body composition assessments

Bone mass (BM in kg), lean tissue mass (LM in kg), and fat mass (FM in kg) of the whole body were assessed using a dual-energy X-ray absorptiometry (DXA Prodigy; GE Lunar Corp., Madison, WI USA) at baseline, 24 months, 36 months, 48 months and 84 months. The coefficient of variation (CV%) of two repeated measurements on the same day was on average 0.7% for BM, 1.0% for LM, and 2.2% for FM in this study.

### Statistical analyses

All data were checked for normality using the Shapiro-Wilk's *W*-test in SPSS 15.0 for Windows. Age-specific means and standard deviations of the anthropometric and body composition traits were calculated. To evaluate if BM, LM and FM track differently from prepuberty to early adulthood, a hierarchical linear model with random effects was employed to explore the growth patterns of height, weight, BM, LM, and FM (*n *= 396 girls). In this model, the time relative to menarche (TRM) was entered as the explanatory variable in the form of polynomial spline functions to explain the change in these variables over time (MLwiN 2.02 software, Multiple Project, Institute of Education, University of London, UK).

To determine how well the girls' BM, LM and FM tracked from baseline to young adult, we compared the within- and between individual variances of these three tissues, adjusting for TRM, compared the proportion of the initial values that remained in the same quartile at the 7-year follow-up visit, and correlated the initial and final measurements during the 7-year period (*n *= 101 girls). Further, comparison of the variances between mother-daughter pairs was assessed using the paired *t*-test (*n *= 144). Spearman's rho correlation was used to evaluate the percentile position for BM, FM, and LM between baseline and the 7-year follow-up.

We used sibling data to estimate the broad sense heritability of body composition with a linear mixed-effects model (*n *= 456 individuals, including brothers and sisters of the girls) [20,21]. In addition, the correlation between daughter (at the age of 18 years) and mother (premenopausal) was used to estimate the maternal effect on the girl's body composition.

To determine what factors were associated with the intra- and inter-individual variation of BM, LM, and FM, we used a generalized estimating equations model of longitudinal data (GEE) with repeated measurements at ages 11 and 18 years. In this report the following predictors of BM, LM and FM of the girls were included in the GEE model: crown-heel length and birth weight, body height and weight, mother's BM, LM and FM, breast feeding, diet index (protein, Ca, K, P, and Mg), LTPA, level of parental education, time of assessment, that is, the baseline at the mean age of 11 and follow-up at the mean age of 18, whether the girl was in the intervention during the first 2 years, and age at menarche.

The *R*^2 ^of the GEE model was computed according to Hardin and Hilbe [22], and it can be interpreted as a similar measure of the proportion of the outcome variance explained by the model in common linear regression. Increments to *R*^2 ^for each regression coefficient were calculated by a formula introduced in Natarajan et al [23]. It is similar to the squared partial correlation computed in linear regression analysis for type III sum of squares and it indicates what proportion of variance explained relates to a given covariate that is unrelated to the other covariates in the model.

The significance of each regression coefficient was tested by comparing the Wald-test statistics for a single degree of freedom with the standard normal distribution. To overcome the problem of collinear predictors, we noted that collinearity is a problem related to the testing of the regression coefficients and not description of data, and subsequently we used two procedures. We used a principal component of collinear covariates measured only at baseline in the GEE model (birth size index = principle component of crown-heel length and birth weight). For covariates that were measured both at baseline and at follow-up, the collinear predictors (diet = Protein, Ca, Mg, K, P; body size = height, weight) were tested as a group using the Wald test statistic for appropriate degrees of freedom (for diet cluster df = 5, body size cluster df = 2 [24]. *R*^2 ^values represent variance explained by the cluster of diet variables, that is, diet index). This was considered useful also from the perspective that individuals do not consume individual nutrients, but combinations of them, so it is their combined explanatory power and statistical significance that is meaningful in this context. For prediction the degree of collinearity should be similar in the target population as observed here [25]. We observed this was the case when comparing the baseline and the follow-up data.

## Results

### Growth patterns

**Figure 1:**
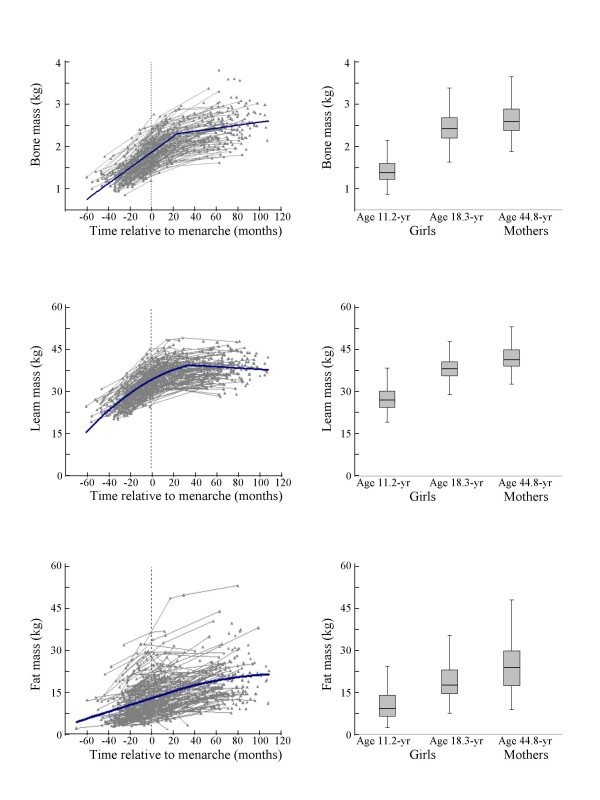
**Growth curves (left panels) for whole body bone mass, lean mass, and fat mass in girls**. The *x*-axis is the time relative to menarche (months). In the left panel, points and thin linking lines indicate individual measurements and the thick line represents the best fitting line estimated by a hierarchical linear model with random effects. In the right panel, each error-box indicates an age group value (mean ± 1 SD shade area). The error line represents the 95% confidence interval.

The growth patterns of BM, LM, and FM, by TRM in the girls are presented in Figure 1. Mothers' BM, LM and FM are presented in Figure 1 for comparison with the values recorded for the girls between 18 and 20 years of age. Height nearly reached the peak value at 3 years post-menarche. Girls at the age of 18 years were similar in height to their mothers (166.1 ± 5.4 vs. 165.2 ± 5.9 cm). In contrast, the body weight of the girls was 9 kg (87%) less than that of their mothers' owing to 0.16 kg (6%) less BM, 3.7 kg (9%) less LM, and more importantly, 5.2 kg (21%) less FM.

### Within and between-individual trait variances

**Figure 2:**
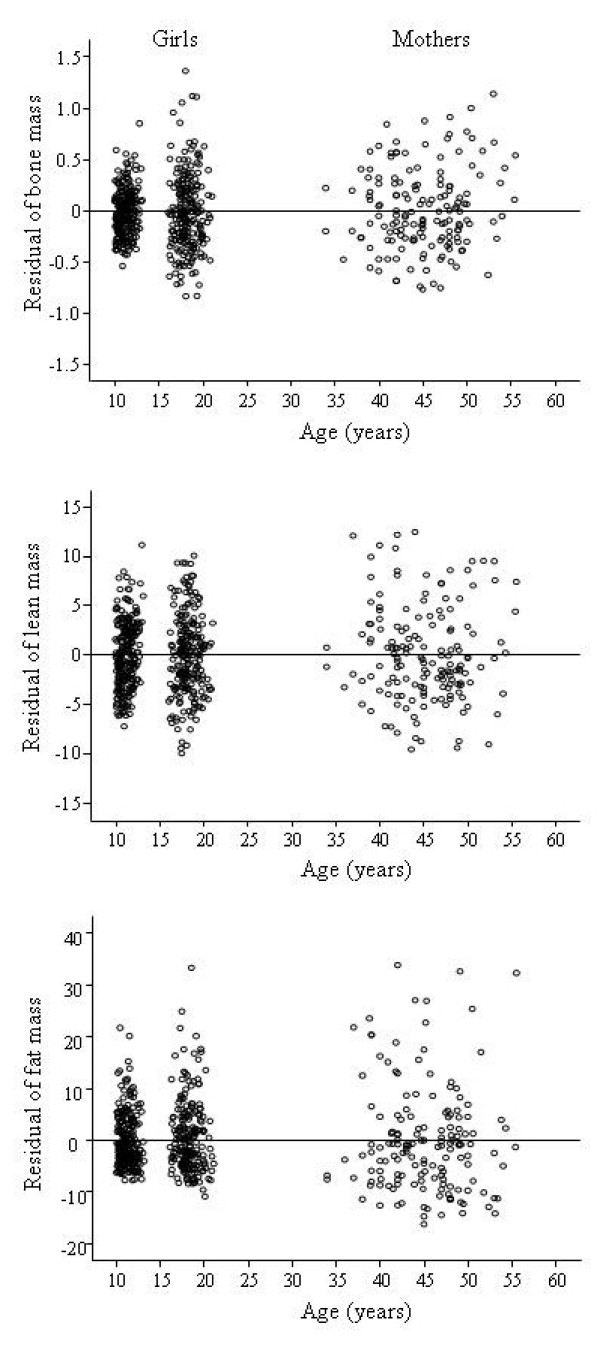
**Residuals of bone mass, lean mass, and fat mass in girls compared with their pre-menopausal mothers**. Each point indicates an individual value. All values are in kg. For girls the value adjusted for time relative to menarche.

During the 7 years of growth, the within-individual trait variances were 14% for height, 18% for weight, 20% for BM, 38% for LM and 24% for FM of the between-individual variances (Table 1, Figure 2). The magnitude of the variance of BM, LM and FM was similar in girls at the age of 18 to that of their mothers in their 40s (Figure 2). The results were similar when we compared only the mother-daughter pairs.

**Table 1:** Total variance of height, weight, and whole body composition traits

			Within-individual variance	Between-individual variance
	Age 11 yr	Age 18 yr	Age 11 to18 yr	Age 11 to 18 yr

	Mean (SD)		Mean (SE)	

Height (cm)	145.6 (8.0)	165.8 (5.0)	4.83 (0.27)	34.5 (2.82)

Weight (kg)	39.2 (8.7)	60.2 (10.0)	12.4 (0.72)	86.4 (5.71)

Bone mass (kg)	1.41 (0.27)	2.46 (0.37)	0.015 (0.001)	0.079 (0.007)

Lean mass (kg)	27.3 (4.3)	38.1 (4.2)	4.28 (0.30)	11.30 (1.06)

Fat mass (kg)	10.6 (5.6)	19.2 (7.4)	8.43 (0.60)	35.0 (3.06)

Mean values and standard deviation (SD) are given. Longitudinal data were separated into variance due to repeated measurements from each individual (within-individual variance) and variance due to difference between individuals. The variance and its standard error (SE) after controlling for time relative to menarche are given.

### Tracking of traits

**Figure 3:**
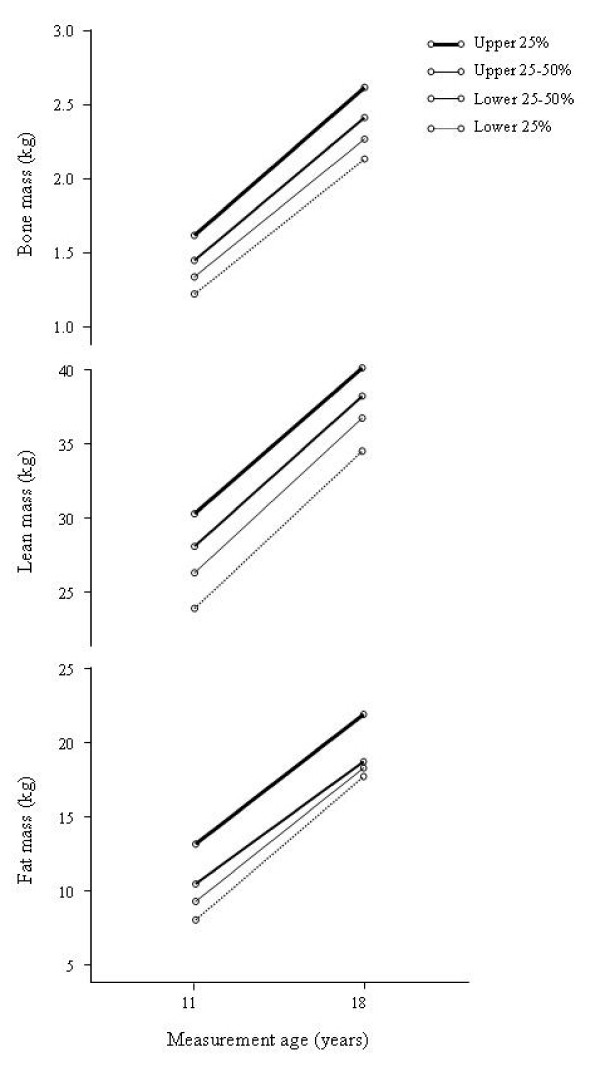
**Estimated marginal means of fat mass, lean mass, and bone mass**. Estimated marginal means of fat mass, lean mass, and bone mass adjusted for menarche age, baseline body height and weight in groups by ranking quartiles at the baseline. Lines with different thicknesses link the baseline and follow-up assessments indicating different quartile groups.

The growth trajectories of BM, LM and FM tracked fairly well from pre puberty to early adulthood. The baseline rank percentile of BM, LM and FM correlated with the follow-up rank percentile 7 years later (*r *= 0.72 for BM, *r *= 0.61 for LM and *r *= 0.65 for FM, all *p *< 0.001). When tracked for decile position, 77% of the individuals remained within their starting decile for BM, 69% for LM, and 66% for FM. To illustrate the tracking of traits, the girls were grouped into quartiles according to the baseline measurements and the four groups increased in parallel (Figure 3). Seventy-nine percent of those in the highest quartile for FM at baseline remained in the highest quartile 7 years later; 73% and 69% in the lowest quartile for BM and LM respectively, remained in their initial quartile 7 years later (Table 2). The girls in the lowest quartile of the BM group matured earlier (age of menarche 13.0 years) than those in the other quartiles (all at age 3.4 years, *p *= 0.032, 0.027 and 0.01, respectively). Similar differences in age of menarche were found in the LM quartiles (lowest vs. 50 to 75% quartile *p *= 0.05, and lowest vs. highest *p *= 0.024). No differences in age of menarche were found between the FM quartile groups.

**Table 2:** The proportion of the subjects in the lowest and highest quartile at both baseline and 7-year follow-up

			Quartile rank at baseline					
			
Cross-table			1–25			75–100		
			
			BM	LM	FM	BM	LM	FM
Quartile rank at 7 years	1–25	BM	73.1			8.3		
			
		LM		69.2			13.0	
			
		FM			51.9			3.7
	
	75–100	BM	3.8			70.8		
			
		LM		0.0			43.5	
			
		FM			0.0			78.9

Mean values and standard deviation (SD) are given. Longitudinal data were separated into variance due to repeated measurements from each individual (within-individual variance) and variance due to difference between individuals. The variance and its standard error (SE) after controlling for time relative to menarche are given.

### Inter-relationships among the traits

The changes in BM, LM and FM correlated positively with each other during the 7-year period (BM with LM r = 0.789, BM with FM r = 0.529, LM with FM r = 0.378, all *p *< 0.001). Sixty-two percent of the variance in BM gain could be explained by the change in LM, while 28% could be explained by the change in FM. There was a negative correlation between TRM and percentage gain in BM (r = -0.537, p < 0.001) and LM (r = -0.622, p < 0.001) but not FM (r = -0.153, p = 0.126).

### Heritability

The heritability in the broad sense was estimated to be 69.3% (heritable component = 56.6%) of total variance of BM, 49.7% (heritable component = 29.5%) of LM, and 57.4% (heritable component = 39.7%) of FM. The correlation between daughter (at age 18) and mother (premenopausal) showed that BM (*r *= 0.439, *p *< 0.001) had the highest correlation coefficient followed by LM (*r *= 0.236, *p *= 0.015) and FM (*r *= 0.187, *p *= 0.054).

### Predictors

In the GEE model, we found that the predictors of BM, LM, and FM in the girls were different (Table 3). The common predictor for high BM, LM, and FM was a high body size index (explaining 62%, 59% and 78% of the variance, respectively), and for high BM and LM and low FM it was diet index (explaining 9%, 24% and 25% of the variance, respectively); for high BM the other predictors were high BM in the girl's mothers (9%), long duration of breast feeding (6%) and low birth size (3%); for high LM another predictor was high LTPA (14%); and for high FM low LTPA (12%). The time-dependent predictors assessed at follow-up more strongly predicted BM, LM and FM than those assessed at baseline (body size for BM, diet and LTPA for LM and FM).

**Table 3:** Generalized estimating equations model accounting for variance in bone mass, lean mass, and fat mass.

Dependent variable	Predictors*	*R*^2^	Main effect *p*-value
Bone mass	Body size index	0.62^†^	< 0.001
	
	Mother's BM	0.09	< 0.001
	
	Diet index	0.09^†^	0.019
	
	Breast feeding	0.06	0.004
	
	Birth size index	-0.03	0.043

Lean mass	Body size index	0.59^†^	< 0.001
	
	Diet index	0.24^†^	< 0.001
	
	LTPA score	0.14	< 0.001

Fat mass	Body size index	0.78^†^	< 0.001
	
	Diet index	0.25^†^	< 0.001
	
	LTPA score	0.12	< 0.001
	
	Parent's education	-0.04	0.036

*The predictors finally entered into the model included assessment times, intervention, age at menarche, birth size index, body size, duration of breast feeding months, LTPA score, diet index, parent's education, and mother's FM, LM, and BM, respectively. Only the significant predictors in the model are shown in the table.

†The *R*^2 ^values for the diet and body size indexes (clusters) are absolute values.

As it is common to have measures of individual nutrients available rather than their combination, we report also the β values for each nutrient in the diet index: BM (β protein = 0.039, Ca = 0.077, Mg = 0.003, K = 0.042, and P = -0.26), LM (β protein = 0.811, Ca = 0.959, Mg = 2.824, K = -0.638, and P = -6.316), and FM (β protein = -1.101, Ca = -1.350, Mg = -2.677, K = 0.615, and P = 7.844). Respectively, for body size index they were: BM (β height = 0.017 and weight = 0.022), LM (β height = 0.395 and weight = 0.181), FM (β height = -0.406 and weight = 0.788).

## Discussion

This study demonstrated that individual differences in BM, LM and FM are established early in life: the variations in the traits seen in adults were already largely expressed in prepubertal girls. The increase in BM was highest near menarche while high LM and FM accumulation occurred much earlier before menarche. Further, we found that the magnitude of the between-individual variance and the individual's relative positions in the lower, middle or upper quartiles for BM, LM, and FM were established before puberty. The ranking of one individual's trait at the baseline remained largely unchanged 7 years later.

The next question was whether these three tissues track differently. Previous studies have reported that 69 to 92% of the variance of bone traits at maturity was accounted for by variances at puberty [26,27]. Total FM, %FM and LM have also been reported to track from childhood to young adulthood [28]. The tracking of total body FM and LM from early childhood into adulthood was low but significant [29,30], whereas the tracking of FM and LM from post-puberty to adulthood was high [28]. In agreement with those studies our data showed that the tracking was stronger in BM than LM and FM during a 7-year follow-up. Further, the correlation between daughters' and their mothers' BM was stronger than that between their LM or FM. In addition, heritability estimated using sibling data also showed that BM was the most heritable trait while LM was the most environmentally modifiable.

In the light of the knowledge that these three tissues tracked differently, it would be important to understand how they are related to each other during growth. We found that the changes in BM, LM and FM correlated positively with each other during the 7-year period, suggesting that change in one tissue may have significant effects on others. In particular, more than 60% of the gain in BM is accounted for by the gain in LM.

Our data together with the results of earlier studies [31,32] suggest that it is possible to identify children who are prone to develop low BM and LM and high FM. The importance of monitoring body composition during puberty resides in the fact that many aspects of body composition during this period are predictive of subsequent measures of these traits in adulthood (that is, body composition 'tracks') [33,34] and, furthermore, are risk factors for many chronic diseases, for example cardiovascular disease, diabetes mellitus, obesity and osteoporosis, in later life [35-38]. Even further, muscle weakness is a risk factor for mobility limitation and disability and risks for falling among older populations [39-41]. LM has been widely used as a surrogate for muscle mass as well as muscle strength. The intra- and inter-individual variability of these three tissues is partly accounted for by a genetic component and partly by environmental factors. Tracking is influenced by both heritable and non-heritable components, both of which need to be taken into account when identifying those at risk for developing low BM and LM and high FM in later life. Interestingly, the girls did not show an increment in LM after age 16. However, by age 18, the girls already had reached 91% of their mother's LM values but only 79% of their mothers FM. This stresses the importance of adolescence in the development not only of BM and FM, but also of muscle mass. The findings on the inter-relationships between the development of LM, BM and FM also suggest that the development of BM, and even FM, during growth can be adjusted by increasing LM through interventions such as increasing physical activity.

Of the modifiable factors, diet is one factor that has been shown to contribute importantly to body composition during adolescence [42,43] and during aging [44]. In an earlier report we showed that supplements of calcium, dairy products and vitamin D did not have a significant effect on bone mass accrual during puberty [11]. In the present study, the intervention did not have significant effects on body composition 4 to 6 years after its conclusion. However, we found that our diet index (a cluster of nutrients) was a strong predictor of BM, LM, and FM. This result clearly underlines the importance of a habitual good diet. All of the nutrients chosen for the model correlated with high milk consumption typical of the Finnish diet and have been reported to have an effect on body composition [12]. In addition, we also found that longer duration of breast feeding was associated with higher BM as has been reported earlier [45,46], and that a high level of parental education was associated with low FM. A higher level of education may be associated with awareness of the importance of a good quality of diet and with a higher income.

Being less physically active may contribute to the larger variation in FM among the post-menarche female population by leading to high FM in early adulthood. Our data, in accordance with data obtained from previous randomized controlled trials [47] and from studies in twins discordant for physical activity during adolescence [48], showed that high LTPA is an independent predictor for low gain in FM. As expected, physical activity was associated with increased LM, as exercise increases muscle mass. Increased use of muscles is linked logically to decreased FM.

The differences in the proportion of within-individual trait variances to between-individual variances in the body composition traits indicates that bone trait variance in the population is largely the result of individual differences in genetic makeup rather than in life style factors [32]. However, this should not be interpreted to mean that an individual's position in the population is immutably fixed. Studies have shown that long-term sports participation during early adolescence results in greater accrual of BM [49-52]. Enhancement of LM seems to be a good predictor of BM accumulation. The effect of physical activity on BM has been shown to be more pronounced in early puberty before menarche [50,52-54], and bone may be more responsive to specific types of physical activity [55-57]. The effects of physical activity on bone are partly attained via muscular activity. An optimal way of inducing bone to respond to physical activity would be to increase its strength without much increasing its total weight. Hence our measure of total bone mass may not be optimal for capturing all the clinically relevant properties of bone as shown by peripheral quantitative computed tomography studies [51,58,59]. It may also be that BM reacts similarly to the increase in physical activity and the gain in body weight during growth.

Although our research approach was rather wide ranging, it has a number of limitations. It is difficult to establish the precise physiological mechanisms that drive the developmental adaptation of human body composition from puberty to early adulthood on account of the array of potentially confounding factors [60]. In our GEE model we did not include hormones, genotypes or fathers' data. Our supplementary heritability estimation used only sibling data, which may affect the estimation of the variance of these three tissues despite the similarity between our estimation and the estimations given in other reports. The high drop-out rate in the follow-up assessments was mainly due to a high proportion of girls going to universities or professional schools and relocating at the end of the follow-up period. However, our statistical methods were able to cope with this since hierarchical models allow inclusion of data from every subject regardless of irregularly-spaced and missing data. The strength of our study is that we were able to use 7-year longitudinal data with multiple factors to assess the whole body composition, including bone, muscle and fat.

## Conclusion

Our data suggest that prepubertal girls with low BM and LM and high FM are prone to develop low values for peak bone and muscle mass and high fat mass in adulthood. A habitual good quality diet is essential for the development of high bone and muscle mass and normal fat mass during puberty. Physical activity is important in increasing muscle mass and is an appropriate strategy for preventing high fat mass during the adolescent growth period.

## Abbreviations

BM: bone mass; Ca: calcium; FM: fat mass; GEE: generalized estimating equations model of longitudinal data; K: potassium; LM: lean mass; LTPA: leisure time physical activity; Mg: magnesium; P: phosphorus; TRM: time relative to menarche.

## Competing interests

The study sponsors played no role in the design, methods, data management or analysis, nor in the decision to publish. The corresponding author had full access to all the data in the study and had final responsibility for the decision to submit for publication. No authors hold any stocks or shares in an organization that may in any way gain or lose financially from the publication of this manuscript. No authors have financial or non-financial competing interests to declare in relation to this manuscript.

## Authors' contributions

SC has full access to all of the data in the study and takes full responsibility for the integrity of the data and for the accuracy of the data analysis. None of the authors have financial or personal interest's affiliations with the sponsors of this research effort. SC, FAT, HK, MA, UMK conceived of and designed the study. SC, EV, AL, SMC, LX, MA were responsible for data acquisition. SC, EV, FAT, AL, TT, LX, SMC, HK, MA, UMK analyzed and interpreted the data. SC, FAT, MA, UMK drafted the manuscript. SC, EV, FAT, AL, TT, LX, SMC, HK, MA, UMK critically revised the manuscript for important intellectual content. SC, EV, FAT, TT provided statistical expertise. SC obtained funding. SC, EV, AL, TT, LX, SMC, HK, MA, UMK provided administrative, technical or material support, and SC, MA, UMK supervised the project. All authors have read and approved the final manuscript.

## Pre-publication history

The pre-publication history for this paper can be accessed here:



## Supplementary Material

Additional file 1 **Appendix 1.** CALEX demographic and health history questionnaire. Click here for file

Additional file 2 **Appendix 2.** CALEX physical activity questionnaire. Click here for file

Additional file 3 **Appendix 3.** Instructions for food diary. Click here for file
