# Supplementary material for: Trait-specific tracking and determinants of body composition: a 7-year follow-up study of pubertal growth in girls
Source: BMC Med. 2009 Jan 26;7:5. doi: 10.1186/1741-7015-7-5 (PMC2639618; doi:10.1186/1741-7015-7-5)
Supplement: Additional file 1 — Appendix 1. CALEX demographic and health history questionnaire. [file 1741-7015-7-5-S1.pdf]

CALEX Demographic and Health History Questionnaire

Study: ______________________________	Date: _________________
Participant Initials: ________	Randomization ID: ☐☐☐☐ ☐☐
Group: ______ Visit: __________	Screening ID: ☐☐☐☐ ☐☐

The following questions ask about the parents.

16. Please check the highest grade of school the parents have completed

Father	Mother
____ Elementary school	____ Elementary school
____ Comprehensive school	____ Comprehensive school
____ Vocational school	____ Vocational school
____ High school	____ High school
____ Professional degree	____ Professional degree
____ Graduate degree	____ Graduate degree
____ Post graduate degree	____ Post graduate degree

17. Does your child live with (please check one)?

____ Both natural parents
____ Mother
____ Father
____ Other, specify: ______________________

18. How well off do you think your family is?

____ Very well off
____ Well off
____ Average
____ Not very well off
____ Not at all well of
