# Supplementary material for: Trait-specific tracking and determinants of body composition: a 7-year follow-up study of pubertal growth in girls
Source: BMC Med. 2009 Jan 26;7:5. doi: 10.1186/1741-7015-7-5 (PMC2639618; doi:10.1186/1741-7015-7-5)
Supplement: Additional file 2 — Appendix 2. CALEX physical activity questionnaire. [file 1741-7015-7-5-S2.pdf]

## CALEX Physical Activity Questionnaire

Participant Initials ☐☐☐ Randomization ID ☐☐☐ Screening ID G ☐☐☐☐

### Physical Activity

1. Outside school classes: How often do you usually exercise in your freetime so much that you get out of breath and swet?

- ☐ Never
- ☐ Less than once a week
- ☐ Once a week
- ☐ 2-3 times a week
- ☐ 4-6 times a week
- ☐ Daily

2. Outside school classes: How many hours a week do you usually exercise in your free time so much that you get out of breath and swet?

- ☐ None
- ☐ About ½ hour
- ☐ About 1 hour
- ☐ About 2-3 hours
- ☐ About 4-6 hours
- ☐ 7 hours or more

3. What kind of physical activities do usually go in for in your freetime? Write three of your favourite physical activities?

In summer: 1)__________________ 2)__________________ 3)__________________

In winter: 1)__________________ 2)__________________ 3)__________________

4. For how long on average, on any single occasion do you participate in this kind of exercise?

In summer:

| 1) | 2) | 3) |
|---|---|---|
| ☐ under 30 minutes | ☐ under 30 minute | ☐ under 30 minutes |
| ☐ 30-60 minutes | ☐ 30-60 minutes | ☐ 30-60 minutes |
| ☐ 1-2 hours | ☐ 1-2 hours | ☐ 1-2 hours |
| ☐ over 2 hours | ☐ over 2 hours | ☐ over 2 hours |

**CALEX Physical Activity Questionnaire**

| Participant Initials | ☐☐☐ | Randomization ID | ☐☐☐ | Screening ID G | ☐☐☐☐ |
|---|---|---|---|---|---|

In winter:

| 1) | 2) | 3) |
|---|---|---|
| ☐ under 30 minutes | ☐ under 30 minute | ☐ under 30 minutes |
| ☐ 30-60 minutes | ☐ 30-60 minutes | ☐ 30-60 minutes |
| ☐ 1-2 hours | ☐ 1-2 hours | ☐ 1-2 hours |
| ☐ over 2 hours | ☐ over 2 hours | ☐ over 2 hours |

5. Are you a member of a sport club?

☐ No
☐ Yes, and I am training in a sports club
☐ Yes, but I don´t participate

**Daily activities**

6. About how many hours per week do you usually spend doing heavy household chores, such as scrubbing floors, vacuuming, sweeping, yardwork, gardening, or snow shoveling?

____ hours per week

During an average 24-hour day, about how many hours do you usually spend sleeping and lying down with your feet up? (Be sure to include time sleeping at night of trying to sleep, resting or stretched out on the sofa watching T.V. etc.)

I usually spend about ____hours a day sleeping and lying down

During an average 24-hour day, about how many hours do you usually spend sitting upright? (Be sure to include time sitting at the table eating, driving or riding in a car of bus, sitting watching T.V etc.)

I usually spend about ____ hours a day sitting upright
