# Supplementary material for: Trait-specific tracking and determinants of body composition: a 7-year follow-up study of pubertal growth in girls
Source: BMC Med. 2009 Jan 26;7:5. doi: 10.1186/1741-7015-7-5 (PMC2639618; doi:10.1186/1741-7015-7-5)
Supplement: Additional file 3 — Appendix 3. Instructions for food diary. [file 1741-7015-7-5-S3.pdf]

CALEX RESEARCH INSTRUCTIONS FOR FOOD DIARY

Please fill the form according the instruction and example. We wish you to have it with you through out the days that you are asked to fill it. We would like to know all the foods and drinks you had had during these days. It will be easier for you to fill it right after eating.

Here are some hints of how to fill the questionnaire:

* Make sure your name and date is on each page
* Write the time of the day when you are eating or drinking.
* Mark the meal you are having using these abbreviation:

BR= breakfast **LN** = lunch **DN**= dinner **SN**= snack

* Write down one drink or food item on one line. You can also use brand names.
* Try to estimate as accurately as possible the portion size. Using food item labels, can size, measuring cups may help. Counting, for example, meatballs or cheese slices you have had. You can also use the pictures of the portions we provided to you.
* Please specify what product or quality the food item is. For example: light berry jogurt or butter compared to margarine, and the brand name of the cheese.
* Remember to record all added things you have had: milk that you have with cereal, butter on bread, sugar in tea.
* Recipes: When having a meal, you can also write down the recipe on how much of each food item it includes.

Please contact us and ask for help if any questions arise!

Name Initial: ______________________
Date: ______________________

School ☐☐☐ ID ☐☐☐☐

FOOD RECORD

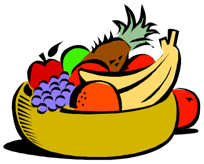

- LIST EVERYTHING YOU HAVE EATEN OR DRUNK DURING THE DAY IN THE RECORD BELOW.
- WRITE ONLY ONE ITEM ON EACH LINE (you don´t have to mark water).
- MARK THE SIZE OF PORTION YOU HAVE HAD AS ACCURATELY AS POSSIBLE

TIME	FOOD OR DRINK	PORTION

- Do you take vitamin or/and mineral supplements (e.g SanaSol, Multi-Tabs)?
  ( ) No ( ) Yes, which product?________________
  How often?_________________________
- Do you follow a special diet ? ( ) No ( ) Yes, which? __________________________
- Do you have lactose-intolerance ? ( ) No ( ) Yes
